# Honesty saves time (and justifications)

**DOI:** 10.3389/fpsyg.2013.00473

**Published:** 2013-07-23

**Authors:** Anna Foerster, Roland Pfister, Constantin Schmidts, David Dignath, Wilfried Kunde

**Affiliations:** ^1^Department of Psychology III, Julius-Maximilians University of WürzburgWürzburg, Germany; ^2^Department of Psychology II, Julius-Maximilians University of WürzburgWürzburg, Germany

The study of active lying poses considerable methodological challenges, especially regarding suitable experimental designs to prompt dishonest responses. This aim is often achieved by instructing participants whether to lie or to be honest in a given situation (e.g., Spence et al., 2001; Walczyk et al., 2003). Shalvi and colleagues have recently promoted a striking alternative approach which allows studying spontaneous lies: the *die under cup paradigm* (Shalvi et al., 2011, 2012; cf. Fischbacher and Heusi, 2008). In this paradigm, participants roll a die, report the outcome anonymously, and receive payment depending on their roll. Though it is not possible to determine whether a given participant is lying or not, the distribution of outcomes indicates whether participants tend to report higher numbers than expected by chance.

Shalvi et al. (2012) modified this paradigm to investigate situational determinants of self-serving lies. In their Experiment 1, participants were to roll a die three times and to report the outcome of the first roll afterward. Importantly, they either worked at their own pace or had to complete the three rolls within 20 s (to induce time pressure). Participants were more honest in the self-paced condition than in the time pressure condition, which led the authors to suggest that lying is an initial, automatic tendency that is overcome only if sufficient time to deliberate is available and if unethical behavior cannot be justified.

These conclusions are surprising because a substantial body of research seems to suggest the very opposite: numerous studies found lying to be cognitively more demanding than responding honestly and, consequently, honesty is typically seen as a behavioral default (e.g., Spence et al., 2001, 2004; Walczyk et al., 2003, 2009; Debey et al., 2012). Further supporting this view, honest answers seem to be actively inhibited during lying (Spence et al., 2001; Nuñez et al., 2005). These findings challenge the interpretations of Shalvi et al. (2012), and we propose that certain peculiarities of the die under cup paradigm are responsible for the diverging results.

As a central feature of the die under cup paradigm, participants can—in principle—generate their response before actually rolling the die. This procedure is markedly different from other approaches where an answer's appropriateness and truthfulness depend on the specific question that is asked in a trial (e.g., Spence et al., 2001). Here, the answer can be generated only after the question is asked. These latter designs might thus be better suited to address the *automaticity* of lying because they tap directly onto the time it takes to generate dishonest and truthful responses. The die under cup paradigm, however, can be modified to address automaticity more directly as well.

Accordingly, we varied the time available for reflection about the (dis)honesty of the reports on two levels: individual die rolls and blocks of rolls. The available time between individual die rolls and reports was manipulated by asking participants either to report the number immediately (*immediate report condition*) or after a short delay (*delayed report condition*). This delay was implemented by asking participants to report only after continuing to shake the cup (Figure 1A). The time available at the level of blocks of rolls was manipulated by having participants run through each condition not only once but repeatedly before and after a short break. Each of these rolls is statistically independent of preceding and subsequent tosses, and hence has always the same expected value. Consequently, any above chance variation of reported numbers over time (i.e., before and after the break) must originate from the human observer. Thus, assessing the time course of reported outcomes provides a novel measure of dishonesty in addition to the mean outcome that has been used previously.

**Figure 1:**
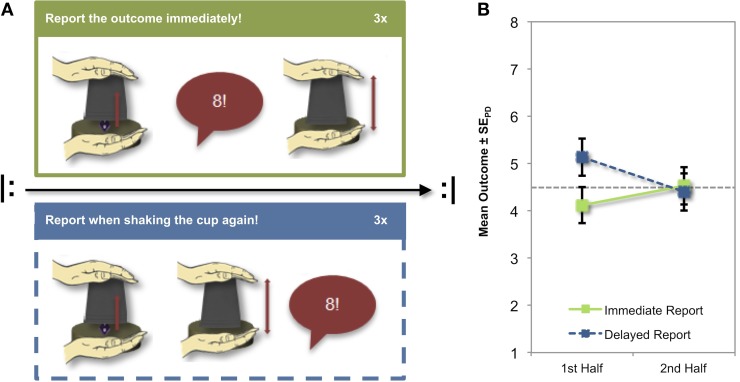
**Design and results. (A)** Procedure of the immediate (top) and the delayed report condition (bottom); the experimenter announced the current condition, and participants reported the outcome of three rolls of an eight-sided die. Both conditions were repeated after a short break. **(B)** In the first half of the experiment, participants reported higher outcomes in the delayed report condition than in the immediate report condition, whereas the conditions did not differ in the second half. Error bars represent standard errors for paired differences, computed separately for each half (Pfister and Janczyk, 2013).

Thirty-two participants (mean age: 24.9 years) joined the game and were informed that they could earn up to 2.50€ depending on the total of 12 rolls with an eight-sided die. They started either with the immediate report condition or the delayed report condition (three rolls) and continued with the remaining condition (three rolls). Crucially, this sequence was repeated after a short break. We had to discard the data of four participants due to procedural errors, leaving data of 28 × 12 = 336 rolls.

Participants reported higher numbers in the delayed report condition than in the immediate report condition in the first experimental half, but not in the second half (see Figure 1B). This observation was confirmed by a 2 × 2 repeated-measures analysis of variance with the factors condition (immediate vs. delayed report) and experimental half (1st vs. 2nd) that was run on the mean outcomes. Most notably, the interaction of condition and experimental half was significant, *F*_(1, 27)_ = 4.96, *p* = 0.034, η^2^_*p*_ = 0.16, whereas neither main effect approached significance; condition: *F*_(1, 27)_ = 2.20, *p* = 0.149, η^2^_*p*_ = 0.08; experimental half: *F* < 1. Tested separately, only the mean outcome of the delayed report condition in the first half differed from chance level (4.5), *t*_(27)_ = 2.28, *p* = 0.031, *d* = 0.43.

These results challenge the interpretations of Shalvi et al. (2012) and suggest spontaneous responses to be quite honest whereas only delayed responses foster self-serving behavior. On a larger timescale, however, time for reasoning (after the first experimental half) seems to counteract dishonest responses again. Thus, dishonest responses do not seem to be a truly automatic tendency but rather do they take more time and cognitive effort than truthful responses. In the light of previous research on lying (Spence et al., 2001; Walczyk et al., 2003) and on spontaneous tendencies to co-operate rather than compete with others (Rand et al., 2012), we thus believe that lying is currently best be seen not as “a dark side of human automatic tendencies” (Shalvi et al., 2012, p. 1269) but rather as the dark side of human *deliberation.*

## References

[B1] Debey E. Verschuere B. Crombez G. (2012). Lying and executive control: an experimental investigation using ego depletion and goal neglect. Acta Psychol. 140, 133–141 10.1016/j.actpsy.2012.03.004 22627157

[B2] Fischbacher U. Heusi F. (2008). Lies in disguise, an experimental study on cheating. TWI Working Paper 40. Thurgau Institute of Economics, University of Konstanz.

[B3] Nuñez J. M. Casey B. J. Egner T. Hare T. Hirsch J. (2005). Intentional false responding shares neural substrates with response conflict and cognitive control. Neuroimage 25, 267–277 15734361 10.1016/j.neuroimage.2004.10.041

[B4] Pfister R. Janczyk M. (2013). Confidence intervals for two sample means: calculation, interpretation, and a few simple rules. Adv. Cogn. Psychol. 9, 74–80 23826038 10.2478/v10053-008-0133-x PMC3699740

[B5] Rand D. G. Greene J. D. Nowak M. A. (2012). Spontaneous giving and calculated greed. Nature 489, 427–430 10.1038/nature11467 22996558

[B6] Shalvi S. Eldar O. Bereby-Meyer Y. (2012). Honesty requires time (and lack of justifications). Psychol. Sci. 23, 1264–1270 10.1177/0956797612443835 22972904

[B7] Shalvi S. Handgraaf M. J. J. De Dreu C. K. W. (2011). Ethical manoeuvring: why people avoid both major and minor lies. Br. J. Manag. 22, S16–S27 10.1111/j.1467-8551.2010.00709.x

[B8] Spence S. A. Farrow T. F. D. Herford A. E. Wilkinson I. D. Zheng Y. Woodruff P. W. R. (2001). Behavioural and functional anatomical correlates of deception in humans. Neuroreport 12, 2849–2853 10.1097/00001756-200109170-00019 11588589

[B9] Spence S. A. Hunter M. D. Farrow T. F. D. Green R. D. Leung D. H. Hughes C. J. (2004). A cognitive neurobiological account of deception: evidence from functional neuroimaging. Philos. Trans. R. Soc. Lond. Ser. B 359, 1755–1762 10.1098/rstb.2004.1555 15590616 PMC1693447

[B10] Walczyk J. J. Mahoney K. T. Doverspike D. Griffith-Ross D. A. (2009). Cognitive lie detection: response time and consistency of answers as cues to deception. J. Bus. Psychol. 24, 33–49 10.1007/s10869-009-9090-8

[B11] Walczyk J. J. Roper K. S. Seemann E. Humphrey A. M. (2003). Cognitive mechanisms underlying lying to questions: response time as a cue to deception. Appl. Cogn. Psychol. 17, 755–774 10.1002/acp.914

